# Temporomandibular treatments are significantly efficient in improving otologic symptoms

**DOI:** 10.1186/s12903-023-03627-2

**Published:** 2023-11-23

**Authors:** Yeganeh Naderi, Elaheh Karami, Goli Chamani, Maryam Amizadeh, Maryam Rad, Mohammad Shabani

**Affiliations:** 1 Oral and Dental Diseases Research Center, Kerman University of Medical Sciences, Kerman, Iran; 2 Department of Oral Medicine and Orofacial Pain, Kerman School of Dentistry, Kerman University of Medical Sciences, Kerman, Iran; 3 Division of Oral Diagnostics and Rehabilitation, Department of Dental Medicine, Scandinavian Center for Orofacial Neuroscience (SCON), Karolinska Institute, Huddinge, Sweden; 4 Clinical Research Development Unit, Shafa Hospital, Kerman University of Medical Sciences, Kerman, Iran; 5 Neuroscience Research Center, Neuropharmacology Institute, Kerman University of Medical Sciences, Kerman, 76198-13159 Iran

**Keywords:** Temporomandibular joint, Otologic, Otalgia, Dizziness, Tinnitus, TMD-therapy, Parafunctional habits

## Abstract

Symptoms of temporomandibular disorders (TMD) could be present as otologic symptoms like earache and dizziness in some patients. In most cases, these symptoms are not recognized because otolaryngologists fail to diagnose TMD as a source of the problem. This investigation was conducted to evaluate the effect of TMD treatments on the otologic symptoms which after taking history and clinical examinations seemed to be related to TMD. In the present study, the patients who were complaining of otalgia, ear fullness, tinnitus, hearing loss, and dizziness were evaluated by an ear fellow. Forty patients who had no known otologic or other primary causes to explain their symptoms, were referred to the orofacial pain clinic with the possible diagnosis of TMD. If the diagnosis was confirmed by an orofacial pain specialist, a combination of TMD treatments was administered to each case and the patients were followed up. The results showed that following implementation of treatment protocols for TMD, more than 50% of the patients reported complete or partial recovery in the second follow-up (p < 0.05). The most common otologic symptom of the referred cases was earache, and the most common associated complaint was neck pain. All the patients had one or more parafunctional habits. This study showed that TMD treatments were significantly efficient in improving otologic symptoms partially or completely and the authors concluded that for the patients with otolaryngologic unexplained symptoms, an overhaul examination is needed to assess TMD as a possible cause of the patient complaint. It is recommended that in cases with unexplained otologic symptoms, otolaryngologists care more about the neck trigger points (TP) and ask about the patient’s parafunctional habits. Otolaryngologists and dentists need to be aware of the risk of developing otologic symptoms caused by these habits or cervical TPs.

## Introduction

Temporomandibular disorders (TMD) refers to a wide range of clinical problems that affect the masticatory muscles, temporomandibular joint, and the surrounding soft tissues (tendons, capsules, ligaments, and connective tissue) [1, 2]. TMD (The abbreviations are detailed in Table 1) is one of the most common causes of chronic orofacial pain of non-dental origin [3–5]. According to a meta-analysis conducted in 2021, the overall prevalence of TMD is approximately 31% in adults and the elderly and 11% in children and adolescents [6]. These disorders are more common in women between the ages of 20 and 40 and appear to increase with age [7–9].

**Table 1:** Comprehensive abbreviation list

Full term form	Abbreviation
Temporomandibular joint	TMJ
Temporomandibular disorders	TMD
Magnetic Resonance Imaging	MRI
Electrocardiogram	ECG
Brief Pain Inventory	BPI-P
Patient Health Questionaire-9	PHQ-9
Anxiety Generalized Disorder-7	GAD-7
Visual Analog Scale	VAS
Oral Behaviors Checklist	OBC
Research diagnostic criteria for TMD	RDC/TMD

TMDs affect either or both articulation and masticatory muscles. The most common signs and symptoms of TMD include temporomandibular joint (TMJ) pain, mouth opening limitation, asymmetric movements of the mandible, pre-auricular and/or masticatory muscle soreness, and joint noises(clicks or crepitus) [10, 11]. TMD also has a significant role in socioeconomic costs, which are frequently linked to comorbidities like depression and other psychological issues [12–14]. The severity of the condition can be assessed using a variety of tools, which can be categorized as anamnestic, clinical, and diagnostic criteria. The most dependable and accurate method for diagnosing TMD is the Diagnostic Criteria for TMD (DC/TMD) [15]. Temporomandibular dysfunction, one of several multifactorial disorders, has primarily been connected to five etiological factors: trauma, intense pain stimuli, parafunctional activities, and psychological components, such as stress, anxiety, and depression [16–18]. However, TMD’s precise etiology is unknown, hence conservative techniques, such as counseling, medication, manual therapy, therapeutic exercise, and occlusal splint therapy, are the favored first-line treatments [19].

In addition, some patients with TMD show ear-related signs and symptoms. Otologic signs and symptoms are less common but have been reported as common TMD signs and symptoms over the last few decades [20]. These signs and symptoms include tinnitus, dizziness, otalgia, burning sensation, decreased or increased hearing, and a feeling of tightness and fullness in the ear [21]. According to epidemiological findings, the prevalence of otologic symptoms in the general population ranges from 10 to 31% and increases to 85% in TMD patients [22]. Studies have shown an association between TMD problems and otologic signs and symptoms [22–24]. Several theories justify the association of otologic symptoms with TMD problems, including anatomical proximity between the TMJ and ear structure, Eustachian tube dysfunction, common embryonic origin, and neuromuscular and neurovascular communication [25]. Most TMD patients can benefit from occlusal splints, acupuncture, correcting parafunctional habits, muscle stretching exercises, physiotherapy, pharmacotherapy, and low-level laser therapy [26, 27].

Some studies have shown that patients with otologic symptoms such as otalgia, dizziness, tinnitus, and ear fullness experience significant or complete improvement after treatment of TMD (17–19). On the other hand, other studies have shown that treatment of TMD is not always effective, and its effect in alleviating otologic symptoms has not been proven [28, 29]. In most cases, patients with otologic symptoms due to TMD, consult an otolaryngologist. Sometimes, due to the lack of diagnosis of the primary problem, they do not receive appropriate treatment, and their symptoms remain for a long time. A systematic review conducted in 2018 stated that due to the limited number of studies conducted in this field and the potential for bias in most studies, there is no sufficient evidence for a positive or negative effect of conservative treatment of TMD in reducing otologic symptoms. Accordingly, there is a need to conduct studies with larger sample sizes and greater precision in different populations. Due to the conflicting results regarding the effect of TMD treatment on reducing symptoms in patients with these disorders, this study aimed to better understand the effect of TMD treatments on otologic symptoms.

## Materials and methods

### Participants

Patients with complaints of earache, tinnitus, hearing loss, and dizziness were referred from an ear fellow to the Department of Orofacial Pain with the possible diagnosis of TMD from January 2021 to January 2022. The Kerman University of Medical Sciences ethics board approved the study, and informed consent was obtained from all the participants. Physical examinations included otolaryngology examinations, cranial nerve examination, stomatognathic examination, and audiometry, MRI and ECG were done in some patients. Patients who had associated systemic diseases with vertigo (like anemia and thyroid issues), as well as patients with earache due to referred pain from the neck and laryngeal masses, were excluded from the study.

If the otolaryngologist did not identify any cause of the otologic symptoms except TMD, the patient was introduced to the Department of Orofacial Pain. Patients who had not received any treatment for TMD by the interview were included in this study. Exclusion criteria were patients who were not compliant with TMD treatment protocol or who did not complete the course of treatment and patients with neurological or cognitive disorders.

### Data collection tools

Before the interviews and clinical examinations, the patients were asked to complete the following questionnaires:

- Brief Pain Inventory (BPI-P) [30].
- Patient Health Questionaire-9 (PHQ-9) [31].
- Anxiety Generalized Disorder-7 (GAD-7) [32].

After completing the questionnaires and gathering the demographic information, including age, marital status, levels of education, medical and medication history, the characteristics of the otologic symptoms, the severity of earache according to the Visual Analog Scale (VAS) [33], and history of exposure to noise pollution were asked.

The history of pain included time of onset of pain, previous history of pain, duration and number of pain episodes, pain quality, pain-aggravating and relieving factors, location of pain, spreading patterns of pain, and previous treatments. Based on the Oral Behaviors Checklist (OBC) [34], a history of parafunctional habits, bruxism (clenching and grinding), head and neck trauma, and jaw locking or dislocation were recorded. Then an orofacial pain specialist examined the patients. Measurement of the mouth opening was done by measuring the distance of the mesioincisal angle of the right upper and lower front teeth. Range of lateral movements relative to the midline, protrusive movements using a millimeter ruler, and jaw deviation or deflection during mouth opening were also recorded. TMJ sound during jaw movements was examined and recorded using a stethoscope.

Tenderness on palpation of the head and neck muscles, including temporalis, masseter (superficial and deep parts), medial pterygoid, splenius capitis, sternocleidomastoid, and trapezius as well as the temporomandibular joint, were assessed. The compression technique of the latent trigger points in the muscles was performed by pressing index finger using a constant, calibrated pressure of 0.5-1 kg/cm2 on the trigger points for at least 2 s. The temporalis muscle was examined at 3 points [35].

**Fig. 1:**
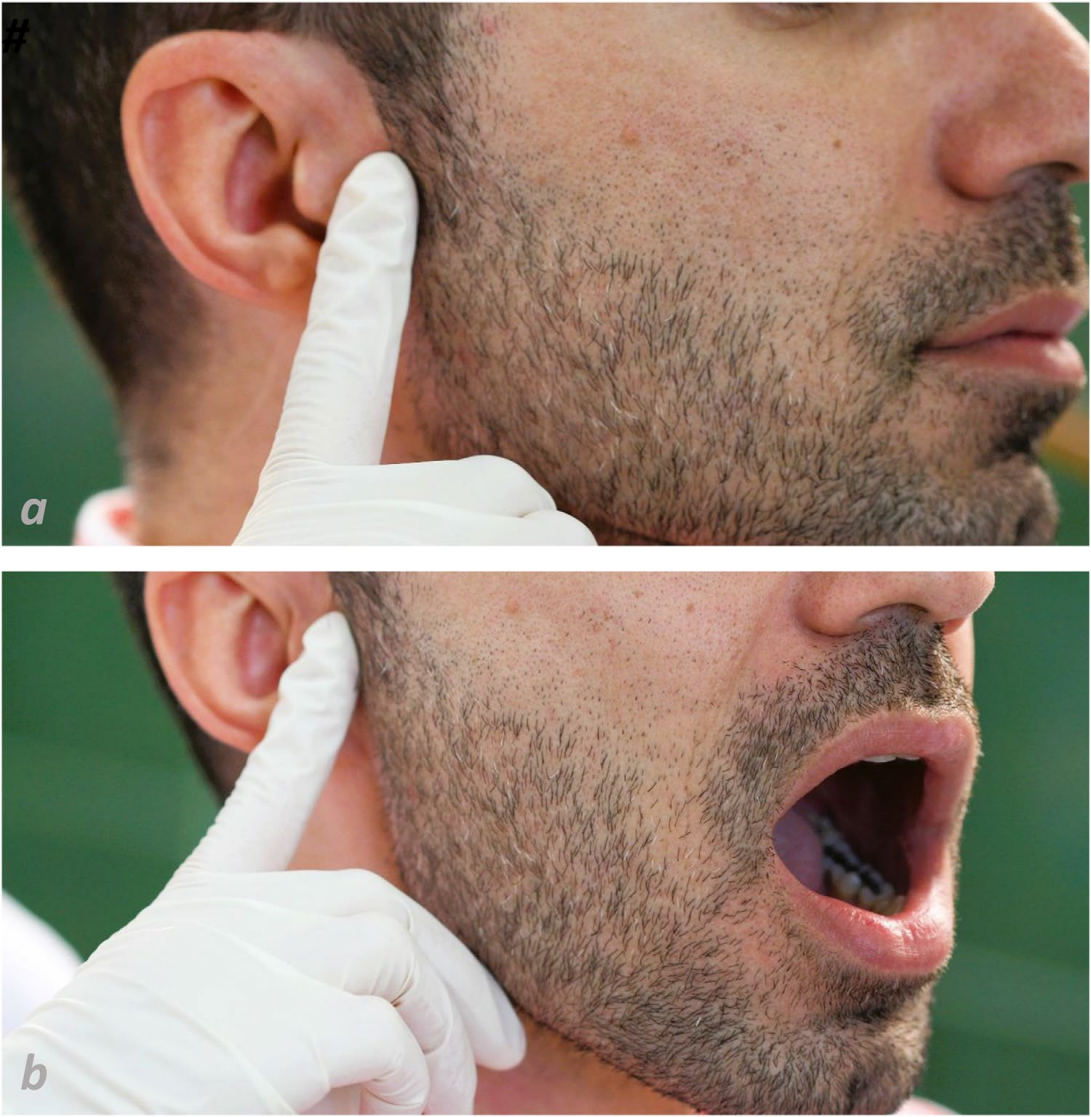
Examination of the lateral pole (**a**) and posterior attachment (**b**) of temporomandibular joint

Pain severity and spreading pattern of pain were recorded. Patients were also evaluated for oral health, dental caries, periodontal disease, tooth wear, abfraction, tooth chipping, cheek ridging, and tongue scalloping. Diagnosis of TMD was based on RDC / TMD (research diagnostic criteria for TMD) (Fig. 1) [36].

### Therapeutic protocol

All patients received standard treatment protocol instructions, including a soft diet, stretching exercises, moist heat and ice pack, correcting neck posture, and eliminating parafunctional habits.

Full coverage stabilizing mandibular night guard was fabricated for patients with parafunctional habits and delivered after two weeks. We ask the patients to abandon unhealthy habits like chewing gum and keep stretching exercises during their follow-up period.

Sleep hygiene tips and proper medication were considered in patients with sleep deprivation or insomnia. Patients were also instructed to do breathing exercises to relieve the stress and tension.

### Therapeutic measures

Two weeks after treatment initiation (including physiotherapy and medication), the patients were asked about the changes in their otologic, and other symptoms attributed to TMD using VAS.

They were also asked to determine the cure rate of the ear symptoms according to the following options: (1) Complete cure, (2) Significant improvement, (3) Relative improvement, (4) No improvement. The patients were also asked to determine the rate of improvement for each otologic symptom. VAS was also used for earache symptoms.

### Data analysis

SPSS 26 program was used for data analysis. First, a descriptive statistic on demographic variables, type of otologic symptoms, and type of treatment of TMD was obtained. Then, Paired T-test was used to determine the rate of improvement over time. A significant value was considered < 0.05.

## Results

In this study, 42 patients were referred from the Department of Otorhinolaryngology to the Department of Oral Medicine and Orofacial Pain. After performing a comprehensive examination to evaluate the temporomandibular joint disorders, 2 patients with the diagnosis of trigeminal neuralgia were excluded based on the exclusion criteria. Finally, 40 patients diagnosed with TMD associated with otologic symptoms were treated. Thirty-five (87.5%) women and five (12.5%) men participated in this study. The mean age of the subjects was 37.16 (SD = 67 13.67).

The prevalence of otologic symptoms is shown in Table 2. Patient examination showed that the most common otologic symptom was earache and the most common associated complaint was neck pain (Table 2). Twenty-nine patients (72.5%) had systemic disease, 27 patients (67.5%) had inappropriate head and neck posture, 6 patients (15%) had a history of noise trauma, 24 patients (60%) had a history of headache with one or a combination of etiologies including TMD attributed headache in 10 (25%), migraine headache in 12 patients (30%) and medication overuse headache (MOH) in 3 patients (7.5%). All patients reported one or more oral parafunctional habits, including clenching, grinding, unilateral chewing, eating enormous amounts of un-shelled seeds, cheek and lip biting, jaw play, chewing gum, nail biting, cheek and lip biting, and lip wetting.

**Table 2:** Prevalence of otologic symptoms

	Otologic symptoms	No.	Pct.
Chief complaint	Otalgia	38	95
	Tinnitus	17	42.5
	Ear fullness	12	30
	Dizziness	11	27.5
	Hearing loss	4	10
	Itchy ear	3	7.5

Signs and symptoms of TMD during patient examinations included capsulitis, jaw click, reduced mouth opening, jaw deviation, myofascial pain, and myalgia in the masticatory muscles (Table 3).

**Table 3:** Prevalence of TMD signs and symptoms

TMD symptoms	No.	Pct.
Capsulitis	34	85
Jaw clicking	18	45
Trismus (limitation of mouth opening)	9	22.5
Jaw deviation	5	12.5
Myofascial pain in masticatory muscles	5	12.5
Myalgia in masticatory muscles	7	17.5
Myofascial pain and myalgia in masticatory muscles	28	70

On examinations of the masticatory muscles, neck muscles, and TMJ, the most imitated otologic symptom in TMD examinations was earache, which was imitated when touching the TMJ, masticatory muscles (superficial masseter, deep masseter, and temporalis) and neck muscles (sternocleidomastoid and splenius capitis). Tinnitus was also imitated when touching the TMJ, the splenius capitis muscle, the superficial masseter, and sternocleidomastoid muscles.

**Fig. 2:**
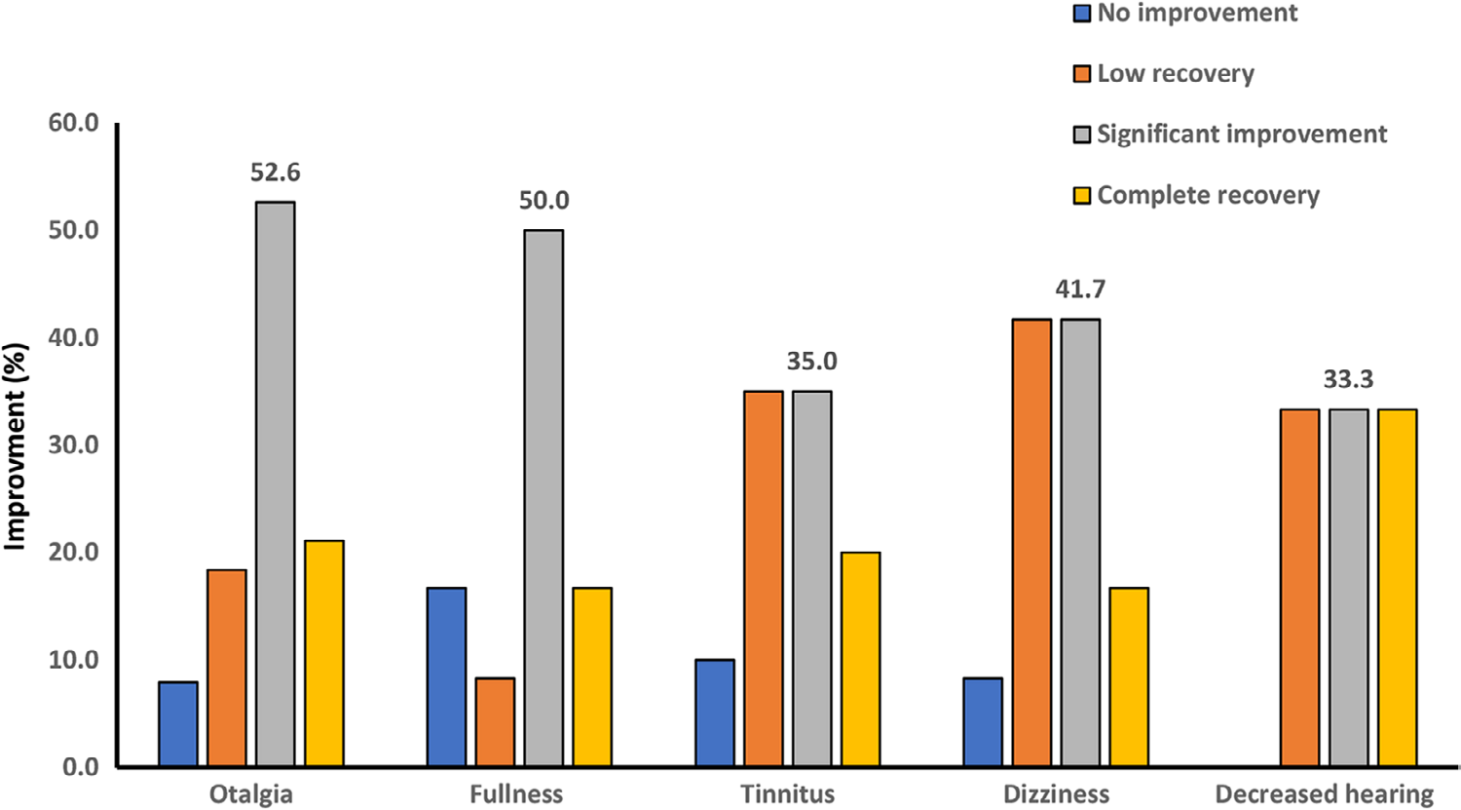
The improvement rate of otologic symptoms after TMD treatment in the first follow-up

**Fig. 3:**
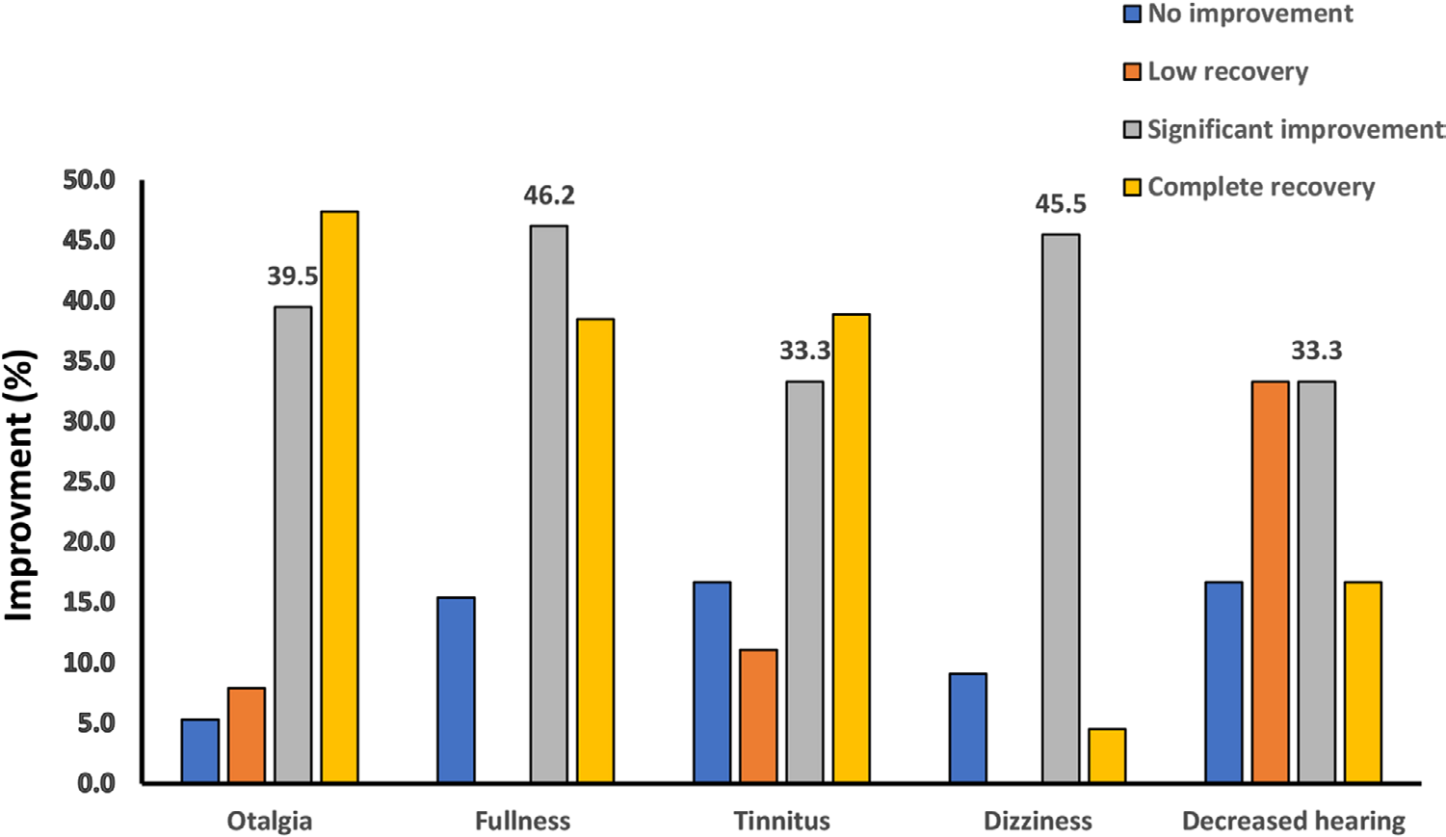
The improvement rate of otologic symptoms after TMD treatment in the second follow-up

**Fig. 4:**
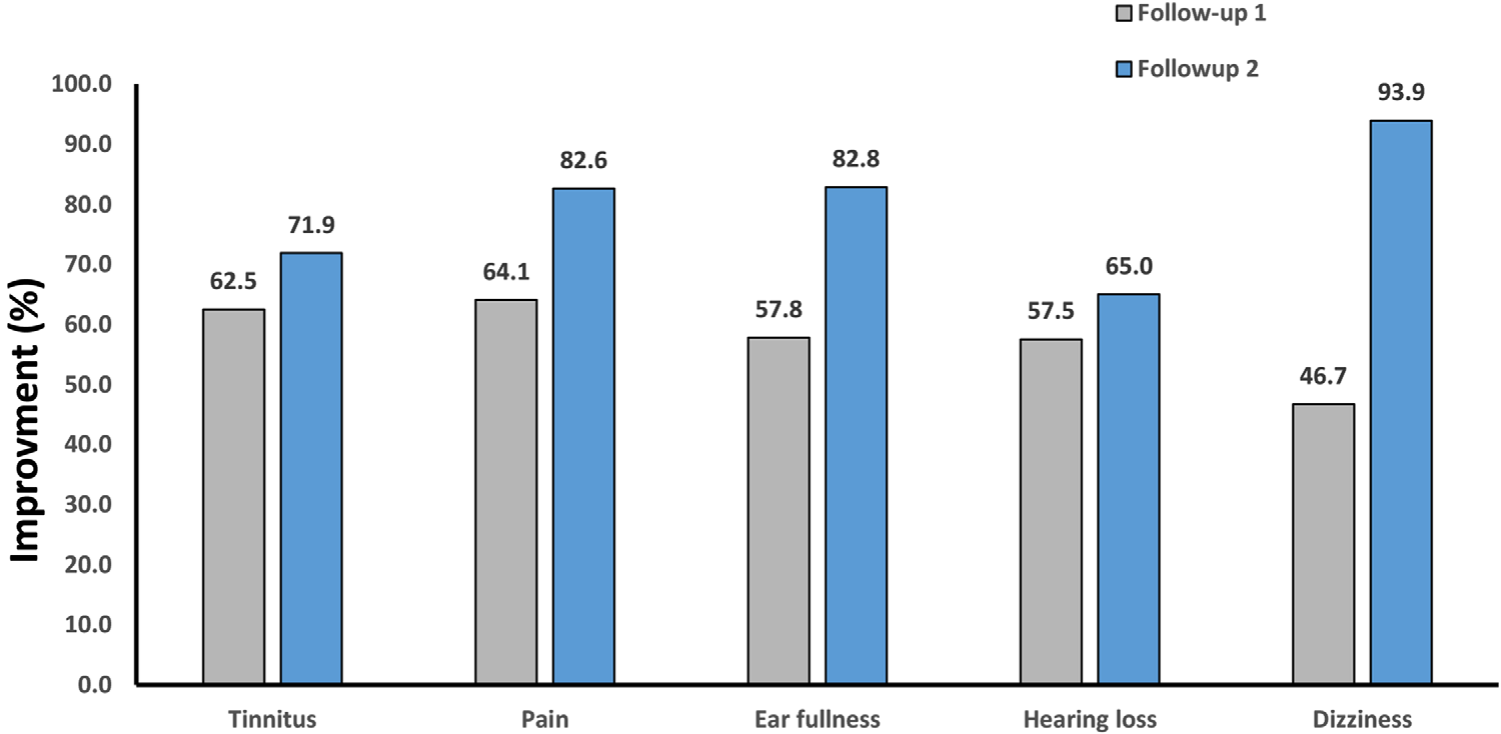
The average percentage of recovery of otologic symptoms based on self-reported patients after TMD treatment. (Follow-up 1: after 2 weeks Follow-up 2: after 2 months)

Patients described the rate of improvement of each of the otologic symptoms at 2 weeks and 2 months’ follow-up. We have reported the results as follows: In the form of complete recovery, significant improvement, low recovery, and no improvement, as shown in Fig. 2, and as a percentage of symptom improvement, based on patients’ self-report according to Figs. 2 and 3. No patient reported exacerbation of the otologic symptoms. Despite the increasing average percentage of recovery in follow-up sessions (Fig. 4) for each of the otologic symptoms, the improvement was significant in the second follow-up (Fig. 3) only for earache (P value = 0.02), ear fullness (0.48), and dizziness (0.05).

According to the BPI questionnaire in the first session, the mean pain intensity was 3.98 out of 10 (SD = 48 2.48), and the mean pain interference with the function was 3.78 out of 10 (SD = 50 2.50). According to the VAS index, the mean pain intensity of patients was initially 45 mm (SD = 0.06 33.06). In the follow-up session, the mean pain intensity was 24.5 mm (SD = 0.01 28.01), which indicated a significant reduction after 2 weeks (p = 0.0001). Also, based on the VAS index the mean of the most severe and lowest pain in the last 5 days and the interference of pain with jaw movements reduced significantly (p = 0.0001).

The GAD and PHQ questionnaires were completed only in the first session. The mean and standard deviation of the GAD and PHO questionnaire were 9.07 (SD = 94 5.94) and 7.94 (SD = 0.2 7.28) showing that most patients had mild anxiety and depression. The results of the mental status assessment based on the VAS index showed improvement in mental status in the follow-up session (P = 0.0001). Twenty-seven (69.2%) patients were sleep-deprived, making it difficult to fall asleep, stay asleep, or both. An assessment of the patient’s sleep quality after 2 weeks based on the VAS index shows an improvement in sleep quality (p = 0.0001).

## Discussion

The present prospective clinical study was conducted to examine TMD treatment’s effect on improving otologic signs and symptoms in patients with TMD. In this study, out of 42 patients who were referred from the ENT department to the Department of Oral Medicine and Orofacial Pain, 17 patients (42.5%) only complained of otologic symptoms.

Numerous studies have been conducted to investigate the association between the prevalence of otologic symptoms and TMD, some of which have found a strong association between the severity of TMD and earache. Patients with moderate to severe TMD often report earache [37–39]. According to epidemiological studies, the prevalence of otologic symptoms in normal individuals ranges from 10 to 31%, although this rate increases to 85% in TMD patients, and 50% of patients who are referred with otologic symptoms do not have otologic problems [40, 41]. According to a systematic review of the effect of conservative TMD treatment on otologic symptoms, there is not enough information to give positive or negative feedback on the symptoms of TMD treatment and most studies are observational and cannot indicate a cause-and-effect relationship [42]. Other meta-analyses showed that using night guards in TMD patients is highly effective in reducing the intensity and frequency of pain. It is also more effective than other treatments or no treatment [43, 44]. In this study, all patients who were wearing night guards had improved earache and dizziness significantly. Although several studies have reported the use of night guard to reduce otologic symptoms, the standard approach (how many hours and for how long a night guard should be used) has not been mentioned. Furthermore, patients have not been examined after treatment [45].

Studies have reported a strong association between TMD and parafunctional habits, including teeth grinding and clenching during sleep with ear symptoms [46, 47]. Parafunctional habits increase the activity of the masticatory muscles, and when these habits exceed the body’s physiological tolerance, they negatively affect the stomatognathic system (mouth and jaw) [48]. In the present study all the patients were advised to abandon parafunction habits (such as teeth clenching and grinding, unilateral chewing, eating seeds, biting cheeks and lips, playing with jaws, chewing gum and nails, and licking or biting lips). Dentists should notify patients of parafunctional habits and inform them of the systemic consequences and the risk of chronic pain due to these unhealthy habits. We found that however two months of follow-up showed that the improvement of otologic symptoms was more than the first follow-up, this improvement was significant only for earache and dizziness symptoms. This result is unreliable for patients with hearing loss due to the small sample size of the patients.

Some studies have shown the relationship between TMD and neck problems due to the relationship between the structure of the mouth and face and the movement of the neck vertebrae and the presence of more trigger points in the neck and shoulders of people with TMD than the general population [49, 50]. Studies have also shown that with the progression of TMD, in addition to otologic symptoms, non-otologic symptoms, the most common of which are headache and back pain, increase [38, 39].

We found that in 16 patients (40%), palpation of splenius capitis and sternocleidomastoid muscles mimicked the otologic symptoms. It is interesting to note that head and body posture play a significant role in developing TMD problems. This is because long-term changes in cranio-cervical position lead to changes in the mandible through mechanical, biomechanical, and neuromuscular mechanisms [50, 51]. Consequently, alternations of head postures, the most usual form of the forward head, and the curvature of the vertebrae resulting from this movement have been considered risk factors in developing muscular TMD. That is why training to correct such incorrect habits for treating TMD, a multifactorial problem, is done by specialists [51, 52].

In this study, 27 patients (67.5%) had inappropriate head and neck positions due to wrong working habits, incorrect positions while studying and using mobile phones, carrying mobile phones in bed, and similar habits. Patients were instructed to correct these habits. In addition, in patients who complained of neck pain or whose neck muscles were painful on palpation and mimicked the patient’s ear symptoms, stretching exercises were performed to deactivate the trigger points of these muscles. Although neck pain was not the main complaint in several patients, palpation of the neck muscles mimicked their otologic symptoms.

Numerous studies in different populations have shown that some types of headaches, including migraines and tension headaches, are more common in TMD patients [53–55]. Some researchers attribute the association between TMD and migraine headaches to their common pathophysiological link through the caudate nucleus of the trigeminal nerve in the brainstem. According to studies, the increasing severity of TMD increases the number of migraine attacks [56, 57]. Patients with TMD and migraine are more likely to develop lateral trigeminal hypertrophy and disc displacement [58, 59].

Although in the present study, headache was the main complaint in only 8 patients (20%), historically, other patients also had experienced headaches such as migraines, headaches attributed to, and Medication overuse headaches (MOH). Patients’ headaches have been replicated by palpation of the muscles such as temporalis and sometimes SCM. Some researchers have suggested a link between the onset of a headache and the stimulation of trigger points in the neck on the same side of the headache [60, 61]. In this study with the onset of TMD treatments, including posture correction and neck muscles stretching, the patients’ headaches got better. Most patients were unaware of the relationship between headache and TMD.

Chronic pain is strongly associated with sleep disorders, both qualitatively and quantitatively. Sleep disorders inactivate analgesic chemical systems and mediators (opioid system, melatonin system, dopamine signaling) and neutralize inflammatory chemical mediators such as Nitric oxide (NO) and adenosine signaling [53]. In this study, 27 patients had poor sleep and the essential recommendations in the form of sleep hygiene were taught to patients. After reviewing the VAS questionnaire, the results showed that after two weeks of sleep, patients improved significantly. According to the results of the present study, conservative treatment of TMD effectively reduces and improves otologic symptoms, especially earache, fullness, and dizziness. In addition, TMD treatment can be effective in improving headache and myalgia.

## Conclusion

Patients with ear symptoms for whom an otolaryngologist cannot determine the specific cause of the symptoms, require a thorough and standard examination to assess the likelihood of TMD as a cause of their symptoms. Anatomical proximity between the TMJ and ear structure, Eustachian tube dysfunction, common embryonic origin, and neuromuscular and neurovascular communication may explain this association. Referral neck pain can also have otologic manifestations even in patients whose neck pain is not one of their main complaints. Therefore, paying attention to myofascial neck pain in patients with unexplained otologic symptoms is important as well. TMD treatments and muscle stretch exercises are effective in completely or partially improvement of unknown otologic signs and symptoms. In addition, due to the limited number of studies on the effect of TMD treatment on otologic symptoms, further studies in this area with appropriate methodology are needed.

### Clinical implications

Many TMD patients with otologic symptoms usually consult with an otolaryngologist but unfortunately mostly receive inappropriate treatment. TMD has multiple etiologies, including muscular, joint, psychological, neurological, and traumatic problems. Accordingly, the treatment also requires a team of specialists such as orofacial pain specialists, psychiatrists, and physiotherapists. As a result, proper conservative treatment of TMD patients with otologic symptoms and timely referral to orofacial pain specialists will prevent confusion, long-term irritation, and chronic problems.

### Limitations and suggestions

During this study, we had to alter our planned data collection and follow-up period because of the COVID-19 pandemic. The number of patients studied in this investigation was limited, and a larger sample size is needed to better assess the effects of TMD treatment on otologic symptoms and draw more accurate conclusions. On the other hand, longer patient follow-up is recommended to assess the stability of alleviation of ear symptoms in TMD patients.

## Data Availability

The datasets used or analyzed during the current study are available from the corresponding author on request.

## Code availability

All software applications used are included in this article.
